# Identification of TIGIT as an HBZ-induced gene by genome-wide analyses: its association with evasion of host defense

**DOI:** 10.1186/1742-4690-12-S1-P56

**Published:** 2015-08-28

**Authors:** Keiko Yasuma, Jun-ichirou Yasunaga, Keiko Takemoto, Kenji Sugata, Norihiro Takenouchi, Masanori Nakagawa, Yutaka Suzuki, Masao Matsuoka

**Affiliations:** 1 Laboratory of Virus Control, Institute for Virus Research, Kyoto University, Kyoto, Japan; 2 Laboratory of Biological Protection, Institute for Virus Research, Kyoto University, Kyoto, Japan; 3 Department of Microbiology, Kansai Medical University, Osaka, Japan; 4 Department of Neurology, Graduate School of Medical Science, Kyoto Prefectural University of Medicine, Kyoto, Japan; 5 Department of Computational Biology, Graduate School of Frontier Sciences, The University of Tokyo, Chiba, Japan

## 

Human T-cell leukemia virus type 1 (HTLV-1) is a causative virus of adult T-cell leukemia (ATL), and inflammatory diseases. HTLV-1 bZIP factor (HBZ) is encoded on the minus strand of HTLV-1, and expressed in all ATL cases. We performed RNA-seq and ChIP-seq using HBZ transduced T cells, and found that expressions of several Treg-related genes, including Foxp3, CD25, CCR4, CCR5, PD-1, NRP1, IKZF family genes, and T-cell immunoglobulin and ITIM domain receptor (Tigit), were upregulated by HBZ. We focused on TIGIT in this study. TIGIT expression was also upregulated in HTLV-1 infected cells in HTLV-1-associated myelopathy/tropical spastic paraparesis (HAM/TSP) patients and CD4+ T cells of ATL patients. HBZ induced the histone modification of the promoter of TIGIT and enhanced its expression. HBZ also enhanced acetylation of H3K18, which is the specific target of p3. HBZ augments transcription of Tigit gene in the presence of PMA/Ionomycin stimulation. Although the detailed mechanism of HBZ mediated activation of Tigit transcription is still unclear, p3 and activated pathway by PMA/Ionomycin is likely involved in its regulation. Furthermore, we analyzed the function of Tigit in HBZ-expressing T cells and CD4+ T cells from HBZ-transgenic mice (HBZ-Tg), and found that stimulation of Tigit with its ligand, PVR, enhanced expression of inhibitory cytokines, such as IL-10, in HBZ-Tg. These results suggest that the enhanced Tigit expression by HBZ may play a key role to modulate the microenvironment where anti-tumor immune response is attenuated by increased expression of IL-10. This environment seems to be associated with the evasion of HTLV-1 infected cells from anti-tumor host immune response and the pathogenesis of HTLV-1 associated inflammatory diseases and ATL.

